# Supplementary material for: Modeling glioblastoma heterogeneity as a dynamic network of cell states
Source: Mol Syst Biol. 2021 Sep 16;17(9):e10105. doi: 10.15252/msb.202010105 (PMC8444284; doi:10.15252/msb.202010105)
Supplement: Supplementary file 5 — Source Data for Figure 3 [file MSB-17-e10105-s001.zip › Figure3A_sourcedata/GSEA_3065/nosigdb_state6.GseaPreranked.1622718443993/DOROSZKO_PERIVASCULAR_INVASION.html]

Details for gene set DOROSZKO\_PERIVASCULAR\_INVASION[GSEA]

|  || Dataset | state6 |
| Phenotype | NoPhenotypeAvailable |
| Upregulated in class | na\_neg |
| GeneSet | DOROSZKO\_PERIVASCULAR\_INVASION |
| Enrichment Score (ES) | -0.6413778 |
| Normalized Enrichment Score (NES) | -2.0727382 |
| Nominal p-value | 0.0 |
| FDR q-value | 6.157895E-5 |
| FWER p-Value | 0.001 |
Table: GSEA Results Summary

  

Fig 1: Enrichment plot: DOROSZKO\_PERIVASCULAR\_INVASION      
 Profile of the Running ES Score & Positions of GeneSet Members on the Rank Ordered List

  

| PROBE | GENE SYMBOL | GENE\_TITLE | RANK IN GENE LIST | RANK METRIC SCORE | RUNNING ES | CORE ENRICHMENT || 1 | PHLDA3 |  |  | 29 | 0.272 | 0.0469 | No |
| 2 | ETV1 |  |  | 196 | 0.177 | 0.0607 | No |
| 3 | GAS7 |  |  | 268 | 0.157 | 0.0817 | No |
| 4 | LMO4 |  |  | 583 | 0.115 | 0.0672 | No |
| 5 | SNX22 |  |  | 627 | 0.112 | 0.0830 | No |
| 6 | MAP2 |  |  | 917 | 0.090 | 0.0667 | No |
| 7 | GNG7 |  |  | 1035 | 0.084 | 0.0688 | No |
| 8 | CKB |  |  | 1359 | 0.069 | 0.0447 | No |
| 9 | MEF2C |  |  | 1785 | 0.054 | 0.0062 | No |
| 10 | MAP1B |  |  | 2893 | 0.024 | -0.1157 | No |
| 11 | S100A6 |  |  | 4326 | -0.005 | -0.2781 | No |
| 12 | PCSK1N |  |  | 4606 | -0.011 | -0.3079 | No |
| 13 | S100A10 |  |  | 5285 | -0.024 | -0.3808 | No |
| 14 | SNX1 |  |  | 6853 | -0.060 | -0.5485 | No |
| 15 | PPFIBP1 |  |  | 6967 | -0.064 | -0.5496 | No |
| 16 | IGFBP5 |  |  | 7011 | -0.065 | -0.5425 | No |
| 17 | RHOB |  |  | 7306 | -0.075 | -0.5623 | No |
| 18 | DMD |  |  | 7992 | -0.107 | -0.6207 | Yes |
| 19 | BCHE |  |  | 8174 | -0.119 | -0.6193 | Yes |
| 20 | PGM2L1 |  |  | 8304 | -0.132 | -0.6096 | Yes |
| 21 | GSN |  |  | 8339 | -0.137 | -0.5881 | Yes |
| 22 | DAG1 |  |  | 8601 | -0.190 | -0.5828 | Yes |
| 23 | ANXA1 |  |  | 8651 | -0.213 | -0.5490 | Yes |
| 24 | VCAN |  |  | 8664 | -0.219 | -0.5099 | Yes |
| 25 | NES |  |  | 8677 | -0.227 | -0.4694 | Yes |
| 26 | COL6A1 |  |  | 8698 | -0.238 | -0.4277 | Yes |
| 27 | IGFBP2 |  |  | 8719 | -0.260 | -0.3820 | Yes |
| 28 | ANXA2 |  |  | 8741 | -0.279 | -0.3329 | Yes |
| 29 | DKK1 |  |  | 8749 | -0.288 | -0.2805 | Yes |
| 30 | PTPRZ1 |  |  | 8783 | -0.360 | -0.2179 | Yes |
| 31 | MEG3 |  |  | 8786 | -0.370 | -0.1496 | Yes |
| 32 | TNC |  |  | 8787 | -0.389 | -0.0778 | Yes |
| 33 | PTN |  |  | 8789 | -0.426 | 0.0008 | Yes |
Table: GSEA details [plain text format]

  

Fig 2: DOROSZKO\_PERIVASCULAR\_INVASION: Random ES distribution      
 Gene set null distribution of ES for **DOROSZKO\_PERIVASCULAR\_INVASION**
